# Diagnosis and treatment of multiple postoperative fistulas following resection of a giant abdominal mesenteric fibromatosis: a case report and literature review

**DOI:** 10.3389/fsurg.2026.1785029

**Published:** 2026-06-02

**Authors:** Weihua Zheng, Junjie Lu, Shuai Jiang, Fan Zhang, Long Xia, Junjing Zhang

**Affiliations:** 1 Foundational and Translational Medical Research Center, Hohhot First Hospital, Hohhot, Inner Mongolia, China; 2 Department of Hepatobiliary Surgery, Hohhot First Hospital, Hohhot, Inner Mongolia, China; 3 Department of Pathology, Hohhot First Hospital, Hohhot, Inner Mongolia, China

**Keywords:** duodenal fistula, mesenteric fibromatosis, multi-disciplinary treatment, ureterorectal fistula, vaginal fistula

## Abstract

Mesenteric fibromatosis (MF) is a rare, locally aggressive, borderline tumor with no metastatic potential, characterized by a high postoperative recurrence rate. This paper reports a complex case of aggressive MF. Despite receiving targeted therapy that was combined with immunotherapy, the tumor in our case progressed rapidly and symptoms of intestinal obstruction developed. Following a challenging radical resection, delayed and complex multiple complications ensued, including duodenal fistula, vaginal fistula, and ureterorectal fistula. Through personalized management under multidisciplinary team (MDT) collaboration—including adequate drainage to control infection, stepwise nutritional support (i.e., transitioning from total parenteral nutrition to enteral nutrition), and targeted management of a high-output enterostomy—the patient ultimately recuperated with drainage tubes in place, and she was able to tolerate oral feeding. This case suggests that for giant mesenteric fibromatosis that continues to progress despite medical therapy, close imaging surveillance (particularly vigilance for signs of "tension cystic change") should be employed to warn of serious complications, and a decisive, surgery-centered multidisciplinary comprehensive treatment strategy should be adopted. Postoperatively, reliance on a multidisciplinary team is essential for long-term vigilance against delayed complex fistulae triggered by factors such as infection. The diagnostic and therapeutic experience from this case provides an important reference for managing this highly challenging disease.

## 1 Introduction

Mesenteric fibromatosis (MF) is a rare clonal proliferative lesion of fibroblasts originating in the mesentery, characterized by invasive growth and a tendency for local recurrence but without metastasis. MF accounts for less than 3% of all soft tissue tumors, with an annual incidence of only 2–4 per million (1), resulting in limited clinical experience in its diagnosis and management. MF has an insidious onset, and its clinical manifestations are mostly related to mass compression, with common symptoms including abdominal pain, abdominal mass, and intestinal obstruction, making early diagnosis difficult. This paper reports a case of a patient with significant gastrointestinal symptoms caused by a giant mesenteric fibromatosis. Despite the extremely high surgical difficulty and complex postoperative complications, the patient survived with a relatively high quality of life following active comprehensive treatment. The diagnostic and therapeutic process of this case will be analyzed and discussed in conjunction with the literature.

## 2 Case presentation

A 31-year-old female patient was admitted on November 23, 2024, due to “abdominal distension, abdominal protrusion for 3 months, accompanied by nausea, vomiting, and poor appetite.” She had no chills, vomited gastric contents, and had a significantly reduced appetite. Past medical history: She had sought medical attention multiple times for recurrent abdominal distension and pain over the past 3 years. In June 2021, she underwent a total colectomy for familial adenomatous polyposis (her rectum was preserved during the operation), followed by stoma reversal in November of the same year. From 2022 onwards, an abdominal mass rapidly increased to approximately 25 cm × 18 cm, resembling a seven-month pregnancy abdomen, causing compression-related difficulty eating. In February 2023, the patient was diagnosed with MF in the middle and lower abdomen at Beijing Cancer Hospital via imaging and biopsy, and she was treated with lenvatinib combined with sintilimab once every 20 days, without surgical treatment. Subsequently, her family took her to several major medical centers in China. However, due to the tumor's tight adhesion to the mesenteric vessels and duodenum, resection could potentially lead to fatal massive hemorrhage or multi-organ failure. Multiple hospitals evaluated and recommended conservative treatment. Her father had a history of mesenteric fibromatosis. Abdominal CT on November 24, 2024 (Figure 1) showed the following: (1) a giant soft tissue mass in the middle and lower abdomen (approximately 248 × 170 × 280 mm) that had significantly enlarged compared with the CT performed at another hospital on September 2, 2024, with expansion of the internal cystic area and increased tension; (2) dilatation and hydronephrosis of the left renal pelvis and ureter; (3) a soft-tissue density lesion in the right abdominal wall, with imaging features consistent with a desmoid tumor; and (4) pelvic and abdominal effusion. Ultrasound-guided puncture drainage of the abdominal cystic lesion was performed following admission, and a follow-up abdominal CT showed that dilatation of the left renal pelvis had significantly improved compared with CT on November 28, 2024, with no residual hydronephrosis or ureteral dilatation.

**Figure 1:**
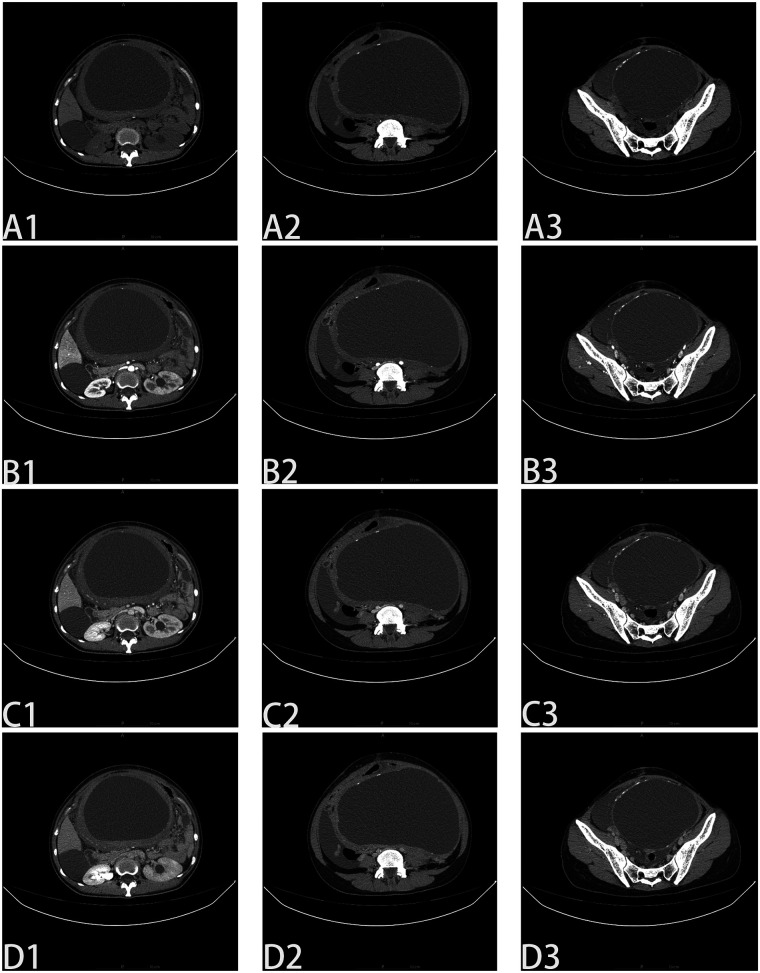
Contrast-enhanced abdominal CT examination. **A1–A3**: A well-defined low-density lesion was observed in the abdomen on plain CT images. We noted no obvious enhancement of the lesion in the arterial phase **(B1–B3)**, venous phase **(C1–C3)**, or delayed phase **(D1–D3)** of contrast-enhanced CT.

The admission diagnoses were (1) mesenteric mass; (2) status post-colectomy; and (3) intestinal obstruction. Upon presentation to our hospital, the patient was critically ill with severe abdominal distension and pain and expressed a strong desire for surgery. Our team, after thorough assessment, decided on a surgical plan involving a Multi-Disciplinary Treatment (MDT) approach in collaboration with the Department of Medical Imaging, Anesthesiology, Interventional Therapy, and Critical Care Medicine. After completion of preoperative preparation, “retroperitoneal lesion resection + mesenteric tumor resection + partial small bowel resection + small bowel enterostomy” was conducted on December 12, 2024 (the surgical procedure was successful, and the postoperative abdominal cavity findings are shown in Figure 2A). It was ascertained that the tumor had invaded a large area of the small intestine during the operation. In order to achieve radical resection, the majority of the small intestine was removed, leaving approximately one meter of residual small intestine postoperatively. The patient developed a high-output stoma after surgery, with a maximum daily output of up to 1,500 mL. In response, we adopted a management strategy that included restriction of oral hypotonic fluids, oral rehydration salts to maintain water and electrolyte balance, and the use of loperamide to reduce intestinal motility and output. During the acute phase complicated by a duodenal fistula, the patient primarily relied on total parenteral nutritional support. After the fistula drainage volume fell, nutritional support was gradually transitioned to a combination of enteral nutrition via nasojejunal tube and parenteral nutrition that ultimately achieved a transition to oral feeding.

**Figure 2:**
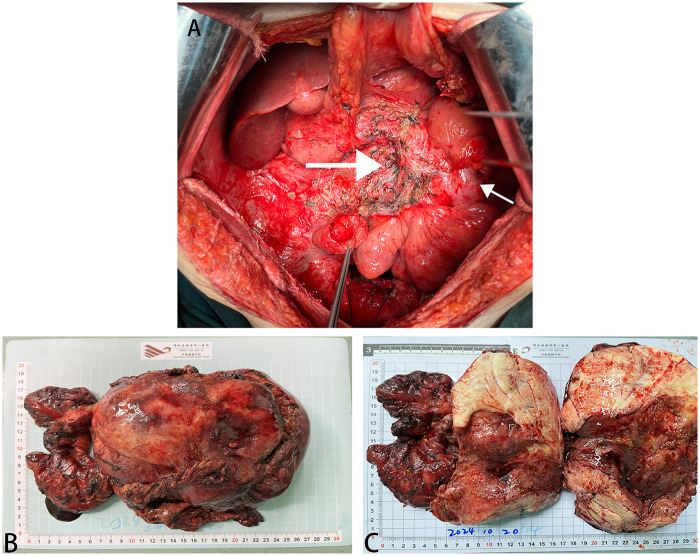
Postoperative abdominal cavity findings and gross specimen of the resected tumor. **(A)** Surgical field after tumor resection (the thick arrow indicates the mesenteric root and the thin arrow indicates the remaining small intestine that is approximately one meter in length); **(B)** Gross specimen of the completely resected tumor; **(C)** Cross-sectional view of the same specimen showing a heterogeneous internal structure and visible necrotic areas.

The postoperative pathological diagnosis was MF, with immunohistochemical results showing cells as CD117 (−), Dog-1 (−), SMA (−), desmin (−), CD34 (−), S-100 (−), and Ki-67 (−)—with negative resection margins (pathologic examination after MF is shown in Figure 3).

**Figure 3:**
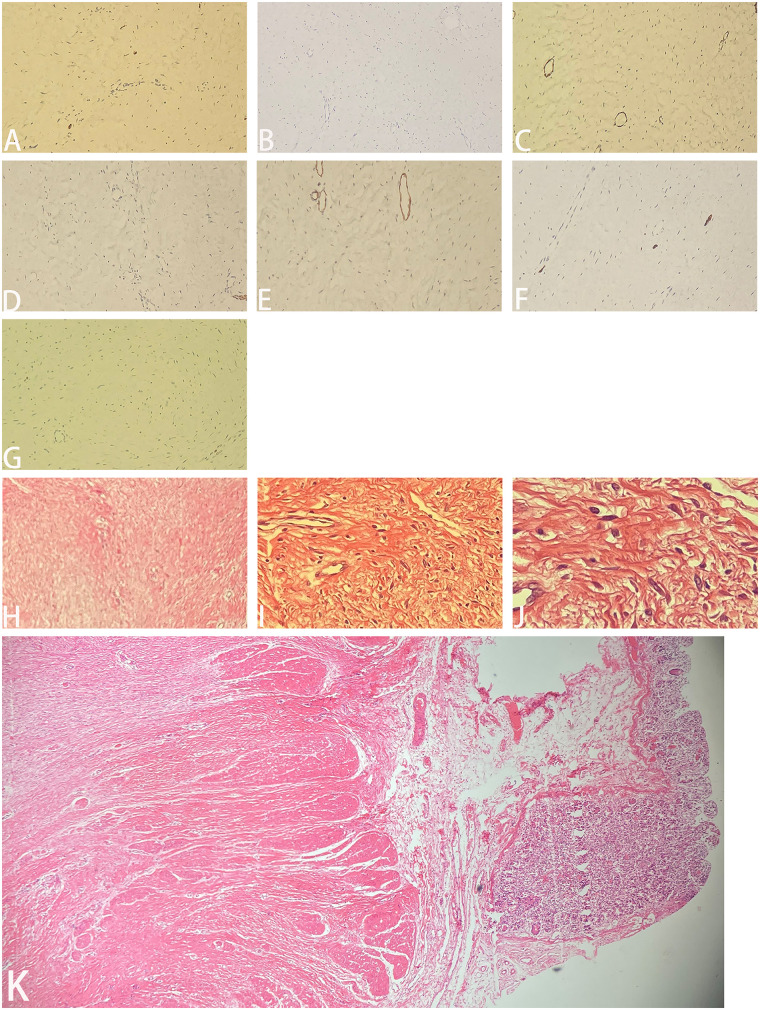
Pathological examination after resection of mesenteric fibromatosis. Immunohistochemical analysis (×200): **(A)** CD117(-), **(B)** Dog-1(-), **(C)** SMA(-), **(D)** Desmin(-), **(E)** CD34(-), **(F)** S-100(-), **(G)** Ki-67(-). **(H)** HE staining (×40), **(I)** HE staining (×100), **(J)** HE staining (×200), **(K)** HE staining (×40). Microscopic examination reveals spindle cell tumor-like proliferation. Combined with immunohistochemical results, the diagnosis is fibroma.

On the 9th postoperative day (December 21, 2024), the patient developed fever (temperature 37.8 °C) and nausea. The drainage fluid from the pelvic and posterior duodenal areas changed from pale yellow to dark red (Figure 4A). Amylase level in the drainage fluid was 2,305.00 U/L, while other markers such as bilirubin were not tested. A follow-up abdominal CT (December 19, 2024) indicated: 1. New abdominal wall lesion, post-partial small bowel resection changes; 2. Right anterior abdominal wall soft tissue lesion smaller than before; 3. Left renal pelvis and ureteral dilatation slightly worsened compared to before; 4. The presence of abdominopelvic effusion accompanied by omental and mesenteric turbidity, with progression compared to the previous examination. Considering the clinical presentation, duodenal fistula and intra-abdominal infection were suspected. Due to the change in condition, the patient requested discharge against medical advice and transferred to a higher-level hospital for further treatment. After 42 days of active treatment at the higher-level hospital, the patient's daily fistula output decreased from 500 mL to 20 mL, and she returned to our hospital for continued nutritional support.

**Figure 4:**
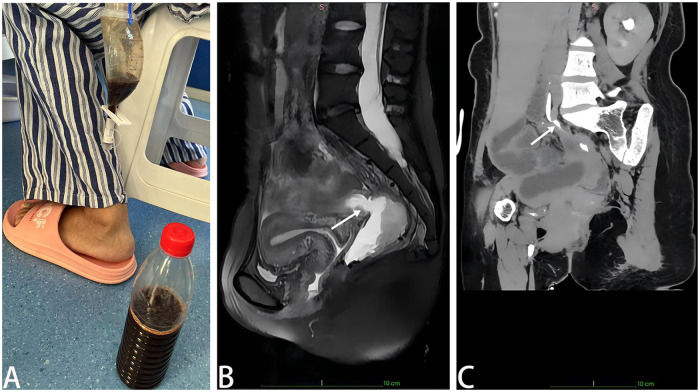
Postoperative complications in the patient. **(A)** The drainage fluid from the pelvic cavity and posterior duodenum changed from light yellow to dark red, suggesting the presence of a duodenal fistula; **(B)** MRI examination revealed a fistula in the posterior vaginal wall that communicated with the pelvic effusion; **(C)** Contrast-enhanced abdominal CT showing contrast agent entering the rectum from the ureter, suggesting the presence of a fistula between the left ureter and the rectum.

On June 15, 2025, the patient was readmitted due to “nausea, vomiting, chills, and high fever for 3 days,” accompanied by abnormal vaginal discharge of approximately 50 mL of yellow secretion. Physical examination revealed lower abdominal tenderness. MR examination (June 17, 2025) (Figure 4B) showed (1) that an abnormal signal tract was observed in the posterior vaginal wall that communicated with the pelvic effusion area, consistent with fistula formation; and (2) that a fluid signal area was visible in the pelvis, with signal characteristics suggestive of fluid and blood accumulation. Due to recurrent fluid collection and infection in the vagina and pelvis caused by the vaginal-rectal fistula, conservative symptomatic treatment was ineffective. A transrectal drainage tube placement under transvaginal ultrasound guidance was performed on July 2, 2025. On July 4, 2025, the patient developed urinary tract irritation symptoms and reduced urine output. Repeat contrast-enhanced abdominal CT examination (Figure 4C) showed (1) postoperative changes after colectomy, abdominal lesion surgery, and partial small bowel resection; (2) the presence of abdominopelvic effusion, with a drainage tube shadow visible in the lower rectum; (3) an abnormal tract between the lower segment of the left ureter and the rectum—with contrast agent entering the rectum from the ureter consistent with fistula formation—with a narrowing of the middle and lower lumen of the left ureter with wall thickening (possibly due to inflammatory changes) and mild dilatation of the upper segment and renal pelvis; and (4) irregular morphology and an unclear boundary of the posterior vaginal wall that corresponded to the fistula location shown on the previous MR (June 17, 2025).During the excretory phase, contrast was seen entering the lower rectum. On that day, 350 mL of yellow turbid fluid was drained from the pelvis, leading to the current consideration of left ureteral fistula combined with pelvic fluid infection and vaginal-rectal fistula. Following MDT consultation, ureteral stent placement was recommended, with possible left nephrostomy if needed. After family discussion, the patient was discharged and sought care at a higher-level hospital. On August 10, 2025, ureteroscopy and left nephrostomy were performed at Peking University People's Hospital, and the patient recovered and was discharged after surgery (the timeline for all clinical diagnoses and treatments is shown in Figure 5). Follow-up is ongoing, and the patient is currently stable, with drainage tubes in place.

**Figure 5:**
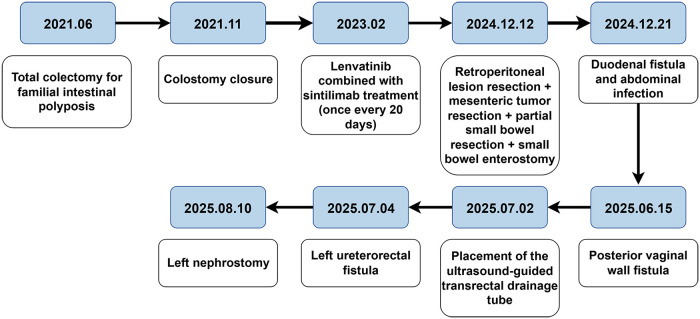
Timeline of clinical diagnosis and treatment.

## 3 Discussion

Mesenteric fibromatosis is a locally aggressive tumor originating from mesenteric fibroblasts. Its exact etiology is not fully understood and is generally believed to involve genetic predisposition, trauma, surgery, and hormonal factors (2, 3). Among these, genetic susceptibility is particularly important, with familial adenomatous polyposis (FAP) being one of its most well-defined genetic predisposing backgrounds. Approximately 10%–15% of FAP patients develop MF (4). This case presented a typical FAP background: the patient's father had a definite history of MF, and the patient herself underwent total colectomy for FAP-related colonic disease. This history strongly supported our contention that the occurrence of MF in this case was related to FAP-associated genetic susceptibility. Further analysis revealed that the clinical manifestations of this case were highly consistent with Gardner syndrome—a specific subtype of FAP accompanied by extraintestinal manifestations such as desmoid tumors. The diagnostic basis included (1) a definite history of FAP; (2) the presence of MF (desmoid tumor), which is a core extraintestinal manifestation; and (3) a first-degree relative affected by MF, supporting an autosomal dominant inheritance pattern. Although the patient had not yet undergone adenomatous polyposis coli (APC) gene testing to confirm the molecular diagnosis and imaging examinations did not reveal other typical manifestations such as osteoma or epidermoid cysts, the above clinical triad (FAP, desmoid tumor, and positive family history) was sufficient to support the clinical diagnosis of Gardner syndrome (5). This diagnosis has important implications for clinical management. For such patients with a definite genetic background we recommend the establishment of a lifelong surveillance system that includes gastroscopy and colonoscopy, regular abdominal imaging examinations (to monitor recurrence or new occurrence of desmoid tumors), and potentially related thyroid screening; while genetic counseling should be provided to family members.

MF often lacks specific clinical manifestations in its early stages and is usually discovered when the tumor enlarges, invades, and encases surrounding intestinal or vascular structures, leading to compression or obstructive symptoms. Therefore, regular imaging follow-up is crucial for monitoring disease progression. In this case, the patient was found to have mid-lower abdominal mesenteric fibromatosis during examination at Beijing Cancer Hospital in February 2023, at which time she had no obvious symptoms and thus began drug therapy. However, during subsequent follow-up, the tumor volume increased significantly within a short period (2 months). Particularly important was that CT imaging suggested “increased tension” in the tumor's internal cystic area, a key imaging warning sign highly suggestive of possible internal hemorrhage or necrosis. The mechanism may be related to rapid tumor growth with relative ischemia in the central region. The continued tumor enlargement directly triggered the patient's intestinal obstruction symptoms. It must be emphasized that without knowledge of the past history, such acute abdominal pain, distension, and signs of peritonitis can easily be confused with common acute abdominal conditions like gastrointestinal perforation or appendicitis, leading to misdiagnosis and delay. When drugs fail to control the disease or signs of obstruction or perforation appear, aggressive surgical intervention is key to saving life, despite the risks of high recurrence rate and organ resection. The successful radical resection in this case, though difficult, provided the patient with a chance for survival.

Treatment strategies for mesenteric fibromatosis include active surveillance, surgery, medication, and radiotherapy, with no unified standard currently. Surgery may achieve a cure but has a high recurrence rate, with some studies reporting postoperative recurrence rates as high as 100% and a median recurrence time of about 28 months (6). Therefore, modern treatment concepts lean more towards active surveillance or drug-first strategies for patients with stable disease. The initial aggressive regimen of lenvatinib combined with sintilimab in this case reflected an attempt at disease control. However, the rapid tumor progression revealed its high biological heterogeneity and the unpredictability of drug response. Ultimately, due to drug ineffectiveness and tumor progression leading to acute intestinal obstruction, treatment was forced to shift from “elective control” to “emergency surgery,” significantly increasing surgical risk and difficulty. This case demonstrates that for mesenteric fibromatosis that progresses rapidly or becomes symptomatic despite drug therapy, a decisive reassessment and active consideration of surgical intervention are necessary to avoid life-threatening complications. In this case, postoperative complications included intra-abdominal infection and significantly elevated amylase in drainage fluid, confirming the occurrence of a duodenal fistula. Subsequently, ureterorectal and vaginal fistulas developed in succession, forming a rare chain of “multiple fistula” complications. These severe complications were first closely related to the locally invasive nature of the tumor, leading to fragile involved tissues. Intraoperatively, the tumor was found to be densely adherent to the horizontal part of the duodenum, with local tissues appearing thin like “eggshell.” Despite the use of staplers supplemented by absorbable suture reinforcement of the seromuscular layer and application of fibrin glue for sealing during surgery, fistula formation was still difficult to avoid. Secondly, extended resection and adhesion dissection inevitably caused mechanical and ischemic injury to adjacent organs. Finally, postoperative intra-abdominal infection could also erode and penetrate the neighboring ureter, rectum, and vaginal wall, forming delayed fistulous tracts. This process warns that when dealing with extensively infiltrating mesenteric fibromatosis, full anticipation of the risk of injury to adjacent organs is necessary. The successful management of this case benefited from two key aspects: first, the correct placement of drainage tubes at critical sites intraoperatively, creating conditions for subsequent management; second, close postoperative monitoring of drainage fluid characteristics and biochemical markers, enabling early identification of complications and providing valuable time for subsequent adequate drainage, anti-infection, and nutritional support therapy.

Postoperative complications such as duodenal fistula further increased the complexity of management. Among such complications, the core issue was maintaining the patient's nutrition and homeostasis, and we therefore adopted a stepwise nutritional support strategy. First, complete parenteral nutrition was used to meet the patient's metabolic requirements while allowing the intestine to rest fully. After preliminary control of abdominal infection and the stabilization of fistula output, enteral nutrition was administered through a nasojejunal tube placed distal to the fistula. The early and appropriate introduction of enteral nutrition facilitated the functional maintenance of the intestinal mucosal barrier and reduced the risk of bacterial translocation. This case highlights the concept that systematic nutritional assessment and active intervention are critically important for patients undergoing major abdominal tumor resection, particularly those who develop severe postoperative complications such as gastrointestinal fistulas. Malnutrition is an independent risk factor for postoperative complications and can significantly increase the risk of infection and anastomotic leakage while delaying recovery (7). Effective nutritional screening tools thus need to be routinely applied during the perioperative period with respect to assessment, and personalized nutritional support regimens should be formulated accordingly. We expect that these tools and regimens will improve the prognosis of complex cases such as ours.

In addition to the acute complications described above, the complexity of nutritional management in our patient was also reflected in the major alterations to intestinal anatomy caused by extensive tumor invasion, as only one meter of small intestine remained after surgery. Although typical short bowel syndrome did not develop, a high-output stoma was generated, as the sharp reduction in intestinal absorptive surface area and altered motility following extensive small bowel resection constituted central challenges to postoperative management. Through stepwise nutritional support (from total parenteral nutrition to combined enteral nutritional support) and targeted pharmacologic management (such as the use of antimotility agents), we successfully maintained the patient's internal homeostasis and promoted intestinal adaptation. Thus, for patients undergoing extensive intestinal resection, the early identification of short-bowel risk, precise management of stoma output, and implementation of personalized nutritional support remain crucial in preventing water-electrolyte imbalance, malnutrition, and related complications. Recent studies have also emphasized systematic perioperative nutritional assessment as a key component in improving patient prognosis.

## 4 Conclusion

The diagnosis and treatment experience regarding this patient indicated that the clinical management of aggressive giant MF needs to adhere to the following key principles. First, emphasis should be placed upon genetic screening to identify and monitor high-risk individuals with a history of familial adenomatous polyposis or desmoid tumors. Second, strict radiologic follow-up should be relied upon, with particular vigilance given toward warning signs such as rapid tumor enlargement within a short period or the appearance of “tension cystic degeneration.” Third, treatment strategies need to remain dynamically adjustable. Once the tumor continues to progress under drug therapy or acute obstruction occurs, the need for radical surgery can be decisively evaluated and ultimately adopted. Finally, the treatment of such diseases is extremely challenging and must rely on an MDT to address complex surgical decision-making and the management of postoperative complications. Only through this comprehensive approach can a balance be achieved between disease control and the preservation of a patient's quality of life.

Although the patient in this case had a complex condition, difficult surgery, and a tortuous postoperative recovery course, through the aforementioned active interventions and management, tumor resection was ultimately achieved. The patient can currently eat orally, and her quality of life has been maintained. This outcome confirms that for complex MF, a comprehensive management pathway of “rigorous monitoring—dynamic decision-making—precise surgery—multidisciplinary support” can achieve a positive balance between disease control and quality of life preservation.

## Ethics and consent statements

This is a retrospective study of one patient. The study was conducted in accordance with the declaration of Helsinki, and all information described in this article is for medical purposes only. Patient information was kept confidential during the study and written informed consent was obtained. This study was approved by the Ethics Review Board of the Hohhot First Hospital.

## Data Availability

The raw data supporting the conclusions of this article will be made available by the authors, without undue reservation.
